# Potential role of *MAP2K1* mutation in the trans-differentiation of interdigitating dendritic cell sarcoma: Case report and literature review

**DOI:** 10.3389/fped.2022.959307

**Published:** 2022-09-16

**Authors:** Alex Jenei, Gábor Bedics, Dániel J. Erdélyi, Judit Müller, Tamás Györke, Csaba Bödör, Ágota Szepesi

**Affiliations:** ^1^Department of Pathology and Experimental Cancer Research, Semmelweis University, Budapest, Hungary; ^2^Hungarian Centre of Excellence for Molecular Medicine - Semmelweis University (HCEMM-SE) Molecular Oncohematology Research Group, Department of Pathology and Experimental Cancer Research, Semmelweis University, Budapest, Hungary; ^3^2nd Department of Pediatrics, Semmelweis University, Budapest, Hungary; ^4^Department of Nuclear Medicine Semmelweis University, Budapest, Hungary

**Keywords:** interdigitating dendritic cell sarcoma, *MAP2K1* mutation, pediatric sarcoma, trans-differentiation, secondary malignant histiocytosis

## Abstract

A 5-year-old male child was diagnosed with interdigitating dendritic cell sarcoma (IDCS) during his maintenance therapy for B-cell precursor acute lymphoblastic leukemia (B-ALL). Multiplex lymph node involvements of the neck were found by positron emission tomography CT (PET-CT). Treatments, including surgical and chemotherapy, resulted in complete remission. Four years later, systemic bone infiltration was discovered. Surgical resection of the IV rib and intensive chemotherapy led to a complete morphological remission, and allogeneic bone marrow transplantation was performed. Comprehensive genomic profiling of the formalin fixed the tumor tissue, and the cryopreserved leukemic cells revealed several common alterations and divergent clonal evolution with a novel *MAP2K1* mutation of the IDCS, which is responsible for the trans-differentiation of the common lymphoid-committed tumor progenitor.

## Introduction

Interdigitating dendritic cell sarcoma (IDCS) is an exceedingly rare dendritic cell neoplasm with even less frequent occurrence among children (1, 2). Based on the recent WHO classification, this entity belongs to the malignant histiocytosis group that also includes histiocytic, Langerhans cell (HS, LCS), and indeterminate cell sarcomas (3). These entities are now considered as “true” hematopoietic tumors arising from bone marrow precursors, together with an L-type histiocytosis, Langerhans cell histiocytosis (LCH), and Erdheim-Chester disease (ECD), while follicular dendritic cell sarcoma shows a different molecular signature similar to the sarcomas of mesenchymal origin (4, 5). IDCS usually affects lymph nodes, whereas extranodal involvement is infrequent (6, 7). While most of the cases are primary tumors, IDCS may also present as secondary histiocytosis, which term applies to cases where histiocytic/dendritic cell tumors follow or appear simultaneously with another hematopoietic tumor. Secondary histiocytic malignancies have been documented following clonal lymphoid proliferations, such as ALL, CLL, follicular lymphoma, hairy cell leukemia, and diffused large B-cell lymphoma (4, 8, 9). The clonal relationship between the lymphoid and the secondary histiocytic tumors has been proven in several cases with IgH and TCR gene rearrangements, or detection of the same mutation or translocation in both tumors (4, 8, 9). However, detailed analyses of molecular pathways involved in the trans-differentiation process are lacking. Here, we present a secondary pediatric IDCS case featuring an activating *MAP2K1* mutation, possibly driving the trans-differentiation of the common lymphoid-committed tumor progenitor.

## Case report

**Figure 1:**
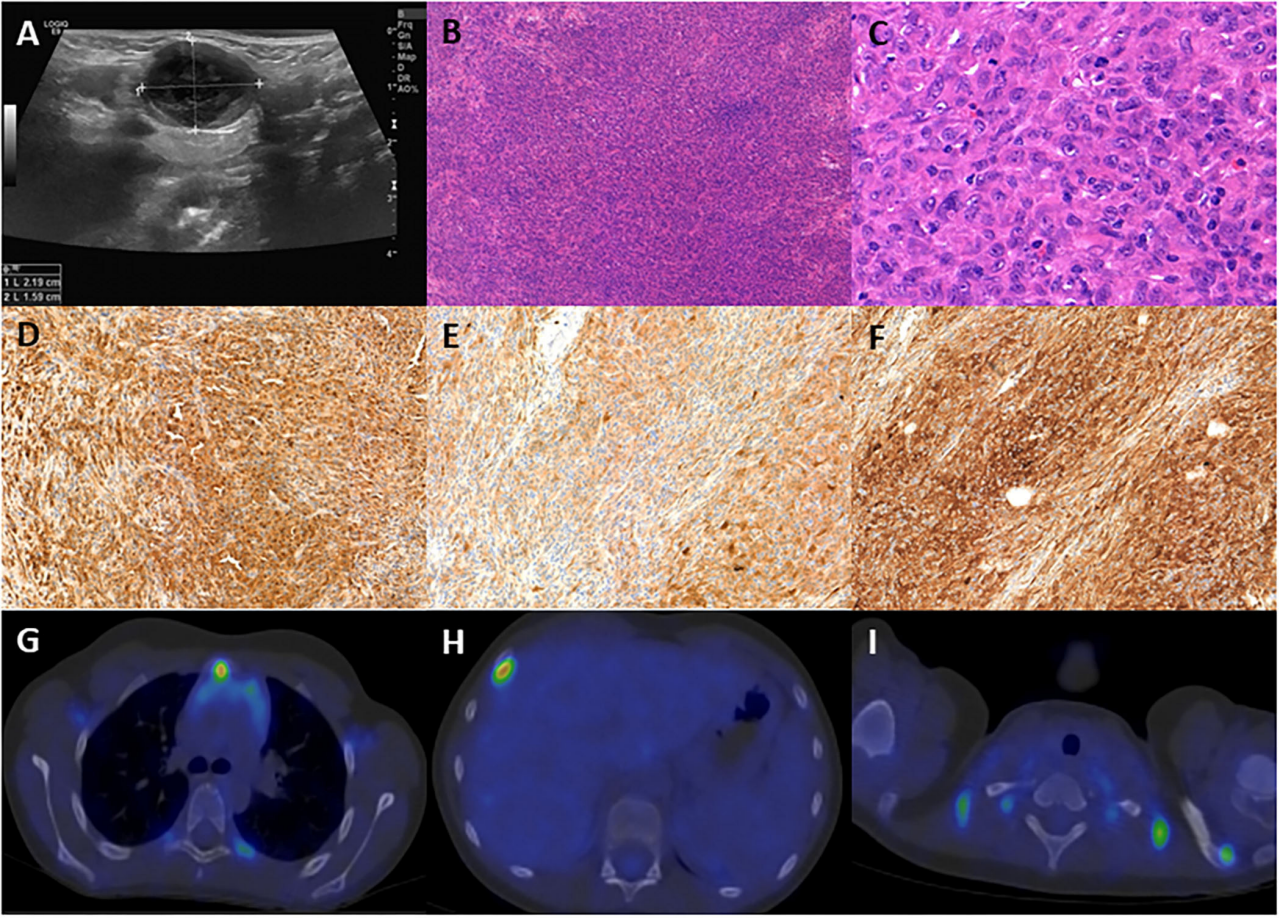
**(A)** Ultrasound picture of the pathologic submandibular lymph node, size: 2.19 × 1.59 cm. **(B)** The excisional biopsy demonstrates diffuse architecture of the lymph node with atypical cell infiltration (100 ×, H&E). **(C)** The lesion is composed of large, elongated or epithelioid cells with vesicular nuclei, multiplex nucleoli, and eosinophilic cytoplasm (400 ×, H&E). **(D)** The neoplastic cells are positive for CD68 (100 ×). **(E)** The neoplastic cells are positive for S100 (100 ×). **(F)** The neoplastic cells are positive for fascin (100 ×). **(G)** PET-CT demonstrates infiltration of the sternal manubrium. **(H)** PET-CT demonstrates infiltration of the 4th right rib. **(I)** PET-CT demonstrates infiltration of the acromial end of the left clavicle.

In June 2016, a 4-year-old boy was diagnosed with B-ALL. He presented with petechiae nosebleed, hepatosplenomegaly, WBC of 128 G/L, Hb of 60 g/L, and platelets of 7 G/L. Flow cytometry detected 86% blasts in the bone marrow aspirate with CD34-, CD38-, CD20-, CD10-, and CD58-positive phenotype, and diploid DNA content, and a 9p21 locus deletion found by fluorescence *in situ* hybridization. He was treated according to the intermediate risk arm of the ALL IC-BFM 2009 protocol. He had good prednisone (PRED) response on day 8. On day 15, 0.7% measurable residual disease (MRD) was detected by flow cytometry. On day 33, his bone marrow became MRD-negative. The parenteral chemotherapy was terminated in January 2017. Subsequently, oral maintenance therapy was initiated. Six months later, a hard, painless lymph node of 2 × 1.4 × 1.4 cm size was discovered in the left submandibular region by ultrasound (Figure 1A). Due to progression with structural irregularities, an open biopsy was performed in December 2017.

Histological examination revealed a spindle cell tumor with the following immunophenotype: LCA, CD68, S100, and fascin positivity and negativity for CD1a, CD21, CD23, CD3, CD20, and CD30 (Figures 1B–F), confirming the diagnosis of IDCS. Bone scintigraphy and bone marrow biopsy excluded bone or marrow involvement. The lymph node excision was followed by cervical block dissection, and histology resulted in multiple lymph nodes involvement by IDCS.

**Figure 2:**
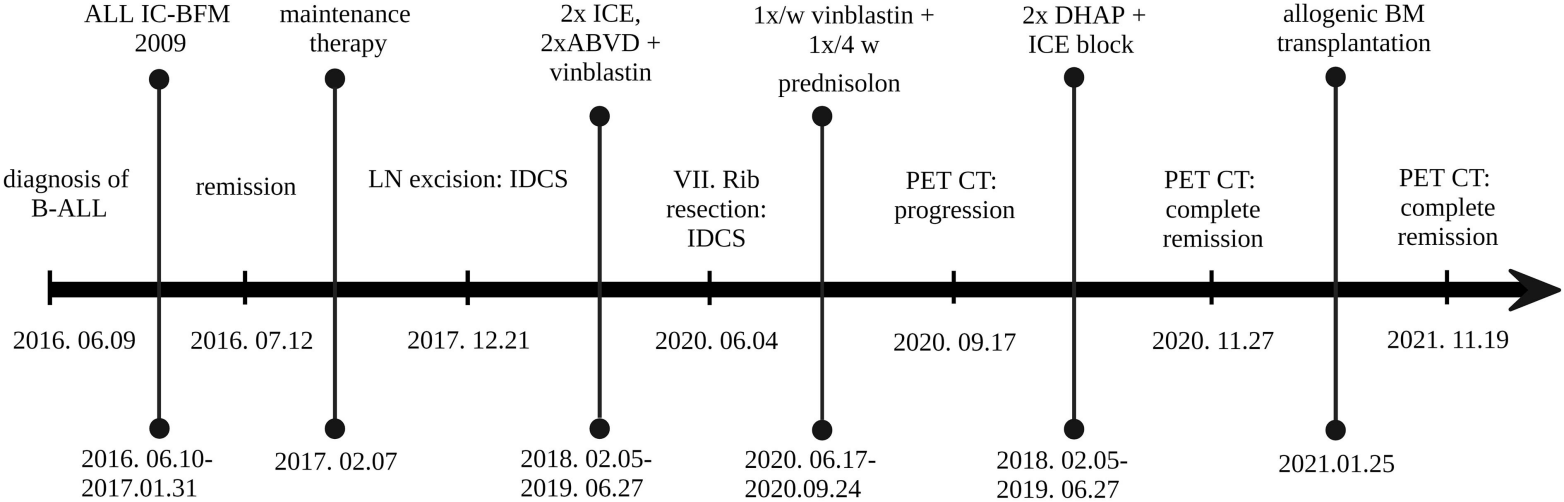
Historical timeline of the case. ABVD, doxorubicin (Adriamycin), bleomycin, vinblastine, dacarbazine; B-ALL, B-lymphoblastic leukemia; BM, bone marrow; DHAP, dexamethasone, cytarabine (Ara C), cisplatin; ICE, infusional ifosfamide carboplatin, etoposide; LN, lymph node. Created with BioRender.com.

The patient received two blocks each of ifosfamide-carboplatin-etoposide (ICE) and adriamycin-bleomycin-vinblastine-dacarbazine (ABVD) chemotherapy, followed by 12 months of oral maintenance with weekly vinblastine (VBL) between June 2018 and June 2019, after which he reached clinical and radiological remission. One year later, multiple enhancing skeletal lesions were identified by the regular follow-up PET-CT (Figures 1G–I). The most intensely affected right IV. rib was resected. IDCS relapse was diagnosed by histology without the presence of residual ALL in the bone marrow. A progressively enlarging cervical lymph node was also resected, although, here, histology indicated only reactive changes. According to the multidisciplinary tumor board decision, from June 2020 to September 2020, he was treated with per os PRED for 5 days/every 4 weeks, combined with weekly VBL. To achieve deep complete remission (CR), his chemotherapy was escalated with two dexamethasone-cisplatin-cytarabine blocks and one more ICE. By November 2020, CR was confirmed by PET-CT. In January 2021, he underwent successful allogenic hematopoietic stem cell transplantation (HSCT). He has remained in CR at the time of this writing (20-month follow-up time), has no identified long-term sequelae, and leads a normal life. He is being followed up by PET-CT and ultrasound scans. The historical timeline of the case is shown in Figure 2.

IgH-gene rearrangement analysis using the Biomed-2 protocol (10) showed identical peaks consistent with the same biallelic or biclonal translocations in the B-ALL and IDCS samples (see Supplementary Figure 1). Comprehensive genomic profiling (CGP) using the Illumina TruSight Oncology500 platform was performed on both IDCS and ALL specimens using a formalin-fixed tumor sample of the lymph node and cryopreserved leukemic cells, respectively (Supplementary material). The CGP found a low mutational burden (4,7 mutation/Mb) in the IDCS specimen. Besides several common alterations mutations of *BTK, SOX17, DOT1L*, and *ATRX* genes were exclusively identified in the B-ALL sample, while a novel activating mutation of *MAP2K1* in exon2 (c.157_171del) was detected only in the IDCS sample (Figure 2, Supplementary Table 1).

## Discussion

Interdigitating dendritic cell sarcoma (IDCS) is a rare aggressive hematopoietic malignancy with poor survival for systemic disease and without standard treatment protocols (6, 11). To date, fewer than 150 cases were reported. Childhood cases represent <10% of them and about 12% of all IDCS cases were presented as secondary neoplasms to other hematopoietic malignancies (6). In 2010, a detailed molecular analysis of LCH and ECD revealed that the proliferation of these tumors is driven by MAP kinase pathway activation (12). Besides the most common mutation of *BRAF*V600E, *MAP2K1* mutation is the second most common alteration found in the *BRAF* wild-type cases (13). Involvement of other components of MAP kinase signaling, e.g., *NRAS, KRAS, ARAF*, and *MAP3K1* mutations, are less frequent and are also described also in the secondary case (14, 15). In patients with systemic histiocytosis, *BRAF* V600E mutation was detected in BM-resident myeloid progenitors, so the cell of origin in these tumors resides in hematopoietic progenitor cells prior to the committed monocyte/macrophage/dendritic cell differentiation (5). In a recent study, beside common *NRAS* and *KRAS* mutations in the hemopoietic malignancies and in the histiocytic tumors, a single case was described with an *MAP2K1* mutation exclusively present in the secondary HS (16). The abundant presence of *IGH*- and *TCR*-rearrangements in sporadic histiocytic tumors indicates that part of these malignancies develop from lymphoid-committed tumor progenitors (17). While recurrent MAPK pathway mutations occur in malignant histiocytosis, e.g., LCS and HS (16, 18, 19), due to its rare occurrence, the molecular background of IDCS is poorly characterized. Recent molecular analyses of few primary and secondary IDCS-cases revealed inactivation of *TP53* (4, 16, 20), while mutation of *SETD2, KMT2D, ERBB3, CDKN2A, MET*, and *SF3B1* and amplification of *c-KIT* and *PDGFR*α were reported in single cases (8, 16, 20). MAPK pathway genes shown to carry activating mutations in IDCS are *NRAS* and *BFAF* (4, 21).

**Figure 3:**
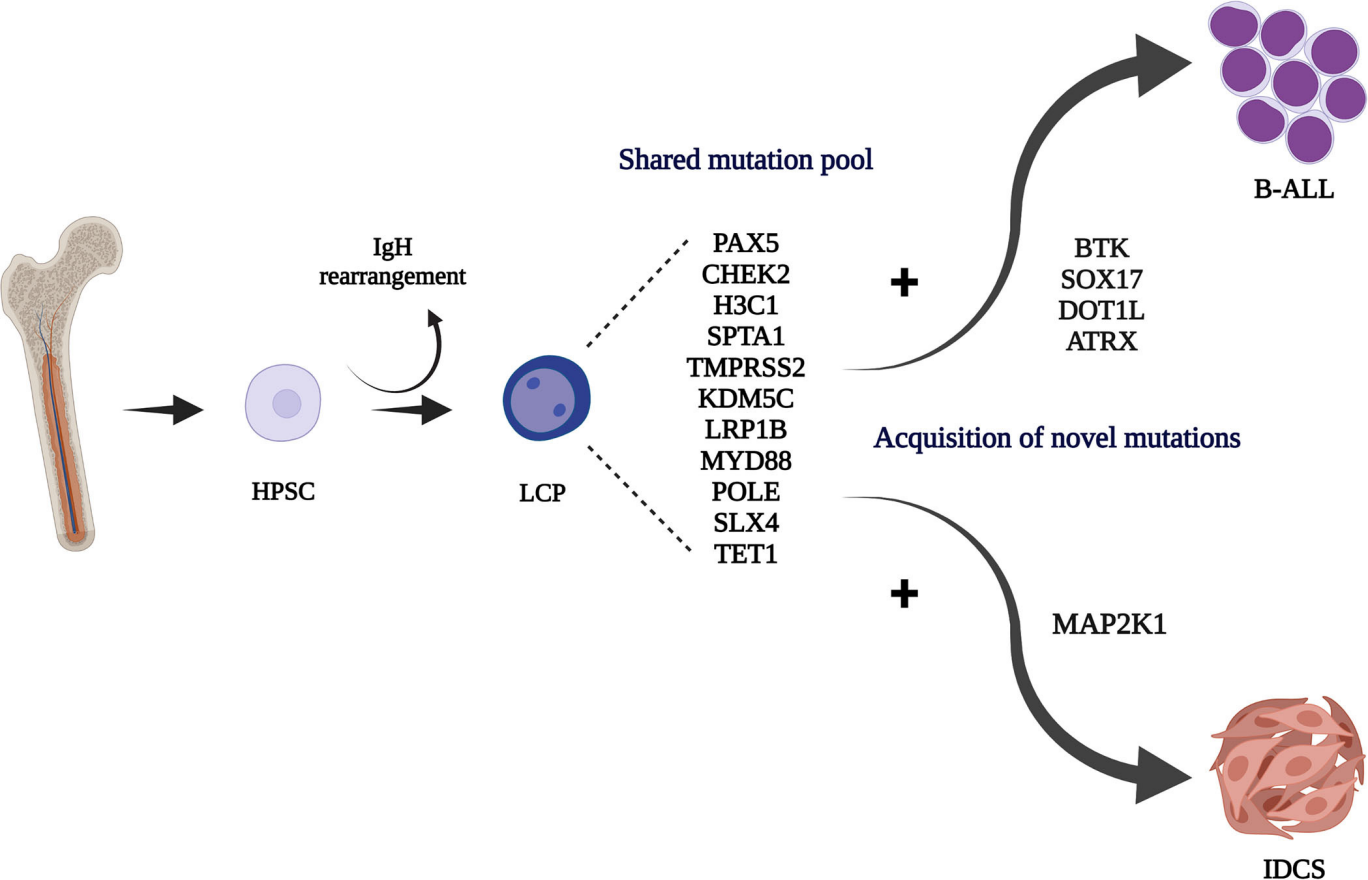
Hematopoietic stem/progenitor cell (HPSC) origin of the IgH rearranged common lymphoid committed tumor progenitor (LCP). Acquisition of different driver mutations may contribute to the development of the B-ALL and IDCS clones. Created with BioRender.com.

Here, we report for the first time a novel *MAP2K1* mutation in a secondary childhood-IDCS case, following B-ALL. Similar activating mutations have been described in 2 LCH cases (c.159_173del) (13, 22). Despite the systemic, relapsing disease, a combined surgical operation and chemotherapy resulted in complete remission, allowing HSCT to offer a final cure (so far, with 20 months of follow-up). This case provides functional evidence linking the cell of origin for this rare malignant dendritic cell tumor to a lymphoid-primed hemopoietic progenitor that could give rise to B-ALL and IDCS by different, specific driver mutations (Figure 3). Involvement of the MAP2K1 pathway in systemic, secondary IDCS tumor development is also confirmed, rationalizing MEK-targeted therapy for refractory cases.

## Data availability statement

The original contributions presented in the study are included in the article/Supplementary materials, further inquiries can be directed to the corresponding author/s.

## Ethics statement

Ethical review and approval was not required for the study on human participants in accordance with the local legislation and institutional requirements. Written informed consent to participate in this study was provided by the participants' legal guardian/next of kin. Written informed consent was obtained from the minor(s)' legal guardian/next of kin for the publication of any potentially identifiable images or data included in this article.

## Author contributions

AJ: collection of the material, data analysis, making the figures, and writing the manuscript. GB: molecular experimental procedures and data analysis. DE and JM: providing clinical data and correction of the manuscript. CB: supervision of experimental procedures, design of pictures, and correction of the manuscript. TG: providing the imaging pictures and correction of the manuscript. ÁS: design of research and writing the manuscript. All authors have contributed to the manuscript, have reviewed and agreed upon the manuscript content.

## Funding

This work was supported by the EU's Horizon 2020 Research and Innovation Program under grant agreement No. 739593, as well as by the Complementary Research Excellence Program of Semmelweis University (EFOP-3.6.3-VEKOP-16-2017-00009) TKP2021-NVA-15, and TKP2021-EGA-24 grants by the Ministry of Innovation and Technology of Hungary from the National Research, Development, and Innovation Fund.

## Conflict of interest

The authors declare that the research was conducted in the absence of any commercial or financial relationships that could be construed as a potential conflict of interest.

## Publisher's note

All claims expressed in this article are solely those of the authors and do not necessarily represent those of their affiliated organizations, or those of the publisher, the editors and the reviewers. Any product that may be evaluated in this article, or claim that may be made by its manufacturer, is not guaranteed or endorsed by the publisher.
