# Supplementary material for: Potential role of MAP2K1 mutation in the trans-differentiation of interdigitating dendritic cell sarcoma: Case report and literature review
Source: Front Pediatr. 2022 Sep 16;10:959307. doi: 10.3389/fped.2022.959307 (PMC9523154; doi:10.3389/fped.2022.959307)
Supplement: Supplementary file 1 [file Table_1.docx]

| Variant_origin | Gene | Chr | Cytoband | Transcript_ID | Variant_type | cDNA_ID | AA_ID | Clinical_Significance |
| --- | --- | --- | --- | --- | --- | --- | --- | --- |
| unique_for_ALL | *BTK* | X | q22.1 | NM_000061.3 | SNV | c.1232G>A | p.G411E | Likely Pathogenic |
| unique_for_ALL | *SOX17* | 8 | q11.23 | NM_022454.4 | Insertion | c.242_243insG | p.D81fs*81 | Uncertain Significance |
| unique_for_ALL | *DOT1L* | 19 | p13.3 | NM_032482.3 | SNV | c.1754T>C | p.L585P | Uncertain Significance |
| unique_for_ALL | *ATRX* | X | q21.1 | NM_000489.6 | SNV | c.6533G>A | p.R2178Q | Uncertain Significance |
| COMMON | *PAX5* | 9 | p13.2 | NM_016734.3 | Insertion | c.963dupC | p.A322fs*19 | Pathogenic |
| COMMON | *CHEK2* | 22 | q12.1 | NM_007194.4 | SNV | c.470T>C | p.I157T | Pathogenic |
| COMMON | *H3C1* | 6 | p22.2 | NM_003529.3 | SNV | c.165T>A | p.Y55* | Likely Pathogenic |
| COMMON | *SPTA1* | 1 | q23.1 | NM_003126.4 | Substitution | c.6793_6794delATinsCC | p.I2265P | Uncertain Significance |
| COMMON | *TMPRSS2* | 21 | q22.3 | NM_005656.4 | Substitution | c.224_225delCAinsTG | p.T75M | Uncertain Significance |
| COMMON | *KDM5C* | X | p11.22 | NM_004187.5 | SNV | c.3533C>T | p.S1178L | Uncertain Significance |
| COMMON | *LRP1B* | 2 | q22.1 | NM_018557.3 | SNV | c.3122A>G | p.N1041S | Uncertain Significance |
| COMMON | *MYD88* | 3 | p22.2 | NM_002468.5 | SNV | c.644+3T>C | - | Uncertain Significance |
| COMMON | *POLE* | 12 | q24.33 | NM_006231.4 | SNV | c.1738C>A | p.H580N | Uncertain Significance |
| COMMON | *SLX4* | 16 | p13.3 | NM_032444.4 | SNV | c.85C>T | p.R29C | Uncertain Significance |
| COMMON | *TET1* | 10 | q21.3 | NM_030625.3 | SNV | c.3062T>C | p.I1021T | Uncertain Significance |
| unique_for_IDCS | *MAP2K1* | 15 | q22.31 | NM_002755.4 | Deletion | c.157_171delTTTCTTACCCAGAAG | p.F53_K57del | Likely Pathogenic |
| unique_for_IDCS | *SDHA* | 5 | p15.33 | NM_004168.4 | Deletion | c.113_115delATG | p.D38del | Uncertain Significance |
| unique_for_IDCS | *BCL10* | 1 | p22.3 | NM_003921.5 | Deletion | c.637_639delGGA | p.G213del | Uncertain Significance |
| unique_for_IDCS | *CREBBP* | 16 | p13.3 | NM_004380.3 | SNV | c.7189C>G | p.Q2397E | Uncertain Significance |
| unique_for_IDCS | *MAP2K1* | 15 | q22.31 | NM_002755.4 | SNV | c.173A>T | p.Q58L | Uncertain Significance |
| unique_for_IDCS | *MCL1* | 1 | q21.3 | NM_021960.5 | SNV | c.799C>T | p.L267F | Uncertain Significance |
| unique_for_IDCS | *SPTA1* | 1 | q23.1 | NM_003126.4 | SNV | c.6190G>A | p.E2064K | Uncertain Significance |

Supplementary Table

Genetic alterations detected by comprehensive genomic profiling in the cryopreserved B-ALL sample and in the formalin fixed IDCS sample.

Abbreviations: ALL: acute lymphoblastic leukaemia; Chr: chromosome; IDCS: indeterminate dendritic cell sarcoma; SNV: single nucleotide variation
