# Supplementary material for: Potential role of MAP2K1 mutation in the trans-differentiation of interdigitating dendritic cell sarcoma: Case report and literature review
Source: Front Pediatr. 2022 Sep 16;10:959307. doi: 10.3389/fped.2022.959307 (PMC9523154; doi:10.3389/fped.2022.959307)
Supplement: Supplementary file 2 [file Data_Sheet_2.docx]

Illumina TruSight Oncology 500 sequencing

**Nucleic Acid Extraction and Quality Assessment**

Genomic DNA and total RNA was isolated from FFPE (formalin-fixed and paraffin-embedded) specimens using the QIAmp DNA FFPE Tissue Kit (QIAGEN GmbH, Hilden, Germany) and the High Pure FFPET RNA Isolation Kit (Roche Diagnostics GmbH, Mannheim, Germany). Estimations of tumor cell percentages of the samples were performed by histopathological examinations prior to the isolation processes. DNA and RNA concentrations were measured using the Qubit dsDNA HS Assay and Qubit RNA HS Assay kits (Life Technologies Corporation, Eugene, Oregon, USA) on the Qubit 4 Fluorometer. 120 ng DNA in 52μl volume was sheared (200 cycles, peak power: 75W, duty factor: 10, treatment time: 510 sec, at 7°C setpoint) using a Covaris E220 Focused-ultrasonicator (Covaris, Woburn, Massachusetts, USA) by the manufacturer’s instructions. The size of double-stranded DNA fragments, and RNA molecules was confirmed after shearing using Tapestation 2200 (Agilent, Cheshire, UK).

**Library preparation and Next-generation sequencing**

Library preparation workflow of Illumina TruSight Oncology 500 High Troughput assay was performed according to the manufacturer’s protocol, briefly: after two hybridization and target-capture steps libraries were amplified (16 cycles [polymerase chain reaction](https://www.sciencedirect.com/topics/medicine-and-dentistry/polymerase-chain-reaction) [PCR]), cleaned, quantified and normalized and pooled. Next-generation sequencing was performed on Illumina NextSeq 2000 platform, with 101 cycles paired-end sequencing.

**Bioinformatic analysis**

Bioinformatic analysis was performed using Illumina TruSight Oncology 500 Local App v2.1. Briefly, raw BCL files were downloaded, FASTQ generation was performed by bcl-convert software. The sequence-alignment to the hg19 reference genome was performed by the Burrows-Wheeler Aligner (BWA-MEM) along with SAMtools utility. Read collapsing analysis was performed in order to remove accurately duplicate reads, marked by unique molecular identifiers (UMIs). Indel realignment and stiching was done by Gemini. Small variant calling was performed by Pisces, which output was filtered by Pepe. Illumina Annontaion Engine Nirvana annotated the small variants using data from COSMIC (v84), ClinVar (2019-02-04), dbSNP (v151), 1000Genomes (Phase 3 v5a), gnomAD (2.1), RefSeq and Ensembl (VEP build 91). Copy number variation calling was performed by CRAFT. For RNA-based analyses each sample was downsampled to 30 million reads, then alignment was performed by STAR to hg19 reference genome and GENCODEv19 reference transcriptome. Duplicates were marked by the Picard duplicate marking algorithm, fusion calling was performed by Manta. Splice variants, tumor mutational burden (TMB) and microsatellite instability were determined by internally developed algorithms. For further clinical interpretation, we used QIAGEN Clinical Insight (QCI) Interpret software, which applies variant filtering and further annotations: pathogenicity scores, population-frequency, protein structure predictions, relevant clinical guidelines and therapeutic options for variants.
