# Supplementary figures and images for: Potential role of MAP2K1 mutation in the trans-differentiation of interdigitating dendritic cell sarcoma: Case report and literature review
Source: Front Pediatr. 2022 Sep 16;10:959307. doi: 10.3389/fped.2022.959307 (PMC9523154; doi:10.3389/fped.2022.959307)

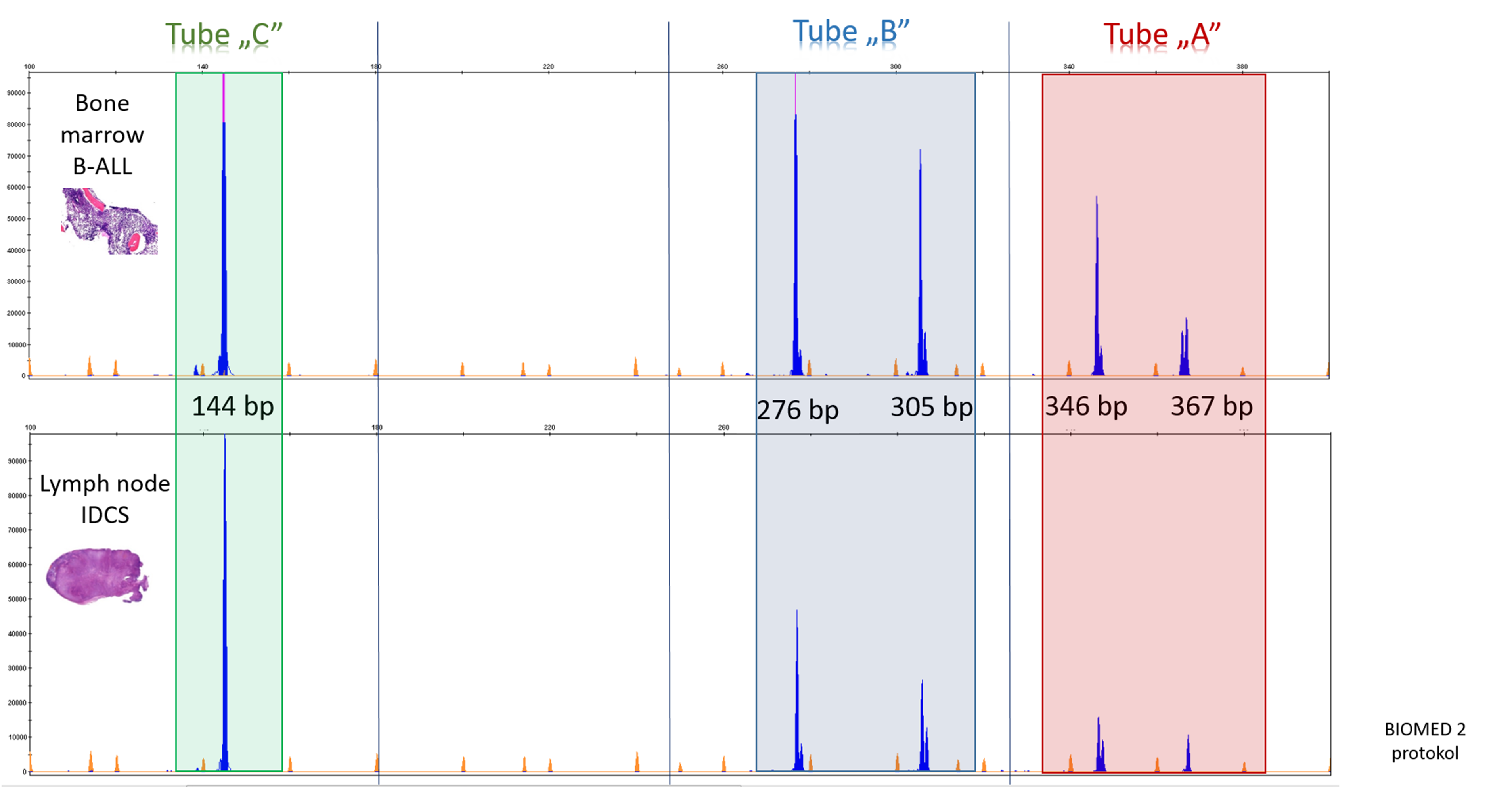

Supplement: Supplementary file 3 [file Image_1.TIF]
